# Single cell cytometry of protein function in RNAi treated cells and in native populations

**DOI:** 10.1186/1471-2121-9-43

**Published:** 2008-08-01

**Authors:** Peter LaPan, Jing Zhang, Jing Pan, Andrew Hill, Steven A Haney

**Affiliations:** 1 Department of Biological Technologies, Section of Biologic Research, Wyeth Research, 87 Cambridge Park Drive, Cambridge, MA 02140, USA; 2 Department of Biological Technologies, Oncology Research, Wyeth Research, 87 Cambridge Park Drive, Cambridge, MA 02140, USA; 3 Department of Biological Technologies, Bioinformatics, Wyeth Research, 87 Cambridge Park Drive, Cambridge, MA 02140, USA; 4 Pfizer Research Technology Center, 620 Memorial Drive, Cambridge, MA 02139

## Abstract

**Background:**

High Content Screening has been shown to improve results of RNAi and other perturbations, however significant intra-sample heterogeneity is common and can complicate some analyses. Single cell cytometry can extract important information from subpopulations within these samples. Such approaches are important for immune cells analyzed by flow cytometry, but have not been broadly available for adherent cells that are critical to the study of solid-tumor cancers and other disease models.

**Results:**

We have directly quantitated the effect of resolving RNAi treatments at the single cell level in experimental systems for both exogenous and endogenous targets. Analyzing the effect of an siRNA that targets GFP at the single cell level permits a stronger measure of the absolute function of the siRNA by gating to eliminate background levels of GFP intensities. Extending these methods to endogenous proteins, we have shown that well-level results of the knockdown of PTEN results in an increase in phospho-S6 levels, but at the single cell level, the correlation reveals the role of other inputs into the pathway. In a third example, reduction of STAT3 levels by siRNA causes an accumulation of cells in the G1 phase of the cell cycle, but does not induce apoptosis or necrosis when compared to control cells that express the same levels of STAT3. In a final example, the effect of reduced p53 levels on increased adriamycin sensitivity for colon carcinoma cells was demonstrated at the whole-well level using siRNA knockdown and in control and untreated cells at the single cell level.

**Conclusion:**

We find that single cell analysis methods are generally applicable to a wide range of experiments in adherent cells using technology that is becoming increasingly available to most laboratories. It is well-suited to emerging models of signaling dysfunction, such as oncogene addition and oncogenic shock. Single cell cytometry can demonstrate effects on cell function for protein levels that differ by as little as 20%. Biological differences that result from changes in protein level or pathway activation state can be modulated directly by RNAi treatment or extracted from the natural variability intrinsic to cells grown under normal culture conditions.

## Background

RNAi has become a widely used method for conducting gene perturbation studies [1,2]. Studies using RNAi to investigate gene function can be highly specific as well as scalable, including whole-genome screens [3-10]. While RNAi can be robust, there are challenges inherent to any RNAi experiment [11,12]. These challenges arise from problems in predicting the specificity of an individual siRNA *a priori*, as well as directly linking the reduced target protein levels with the observed effects [13,14]. Despite these challenges, RNAi is the most versatile and robust method for broadly testing gene function in most eukaryotes [15].

High content screening (HCS), or automated quantitative immunofluorescence, is being used to an increasing extent in the target validation stage of drug development, as well as in basic science [16,17]. Image analysis is used to identify, quantitate and track multiple measures of individual cells [18-20]. Usually, these data are averaged, which is analogous to whole-well assays such as caspase activity or reporter gene expression. The advantage of HCS even in analyses at the whole-well level is that cells can be individually screened for inclusion in the well average according to parameters such as the health of the cell, stage in the cell cycle or activation state of a signaling pathway.

Single cell cytometry (or single cell analysis) has been used historically to analyze complex populations of cells, such as the study of differentiating immune cells by flow cytometry [21,22]. Recently, the use of flow cytometry and single cell analysis has been applied to signaling pathways within cancer cell lines [23-26]. These studies highlight two advantages to flow cytometry-based single cell analysis. First, the ability to integrate the study of more than one cell-signaling pathway into an assay allows the classification of cancer cells according to perturbation responses, rather than static pathway activation levels. This better recapitulates the complex stimuli cancer cells encounter *in vivo*. Furthermore, advanced solid-tumor cancers are comprised of multiple subpopulations of cells, based on their genetic fluctuations and their interactions with host cells and tissues. Single cell analysis is capable of measuring changes within each of these subpopulations [25,27-29]. The methods developed to analyze interrelationships between thousands of data points in each of multiple samples are advancing biological and pharmaceutical research beyond the study of single pathways, and towards the study of outcomes that arise from complex interactions between multiple pathways [24,30,31]. Such approaches are gaining favor because single-pathway studies show only limited correlations across cell lines or clinical samples, whereas the integration of multiple pathways and over complex sets of stimuli, enable more accurate understandings of cell signaling by addressing direct signaling as well as cross-pathway regulation [32].

We have used HCS to characterize the effects of genetic and chemical perturbations on cells by single cell analysis. We find that the wide range of protein expression levels in unperturbed cells is a significant complication for RNAi experiments, but that this complication can be addressed directly by analyzing such experiments at the single cell level. These methods allow the study of protein function by measuring the response in distinct subpopulations of cells in culture that result from stochastic variability of a target protein in a culture of cells.

## Results

### Analysis of RNAi-mediated knockdown of GFP at the whole-well and single cell levels

**Figure 1:**
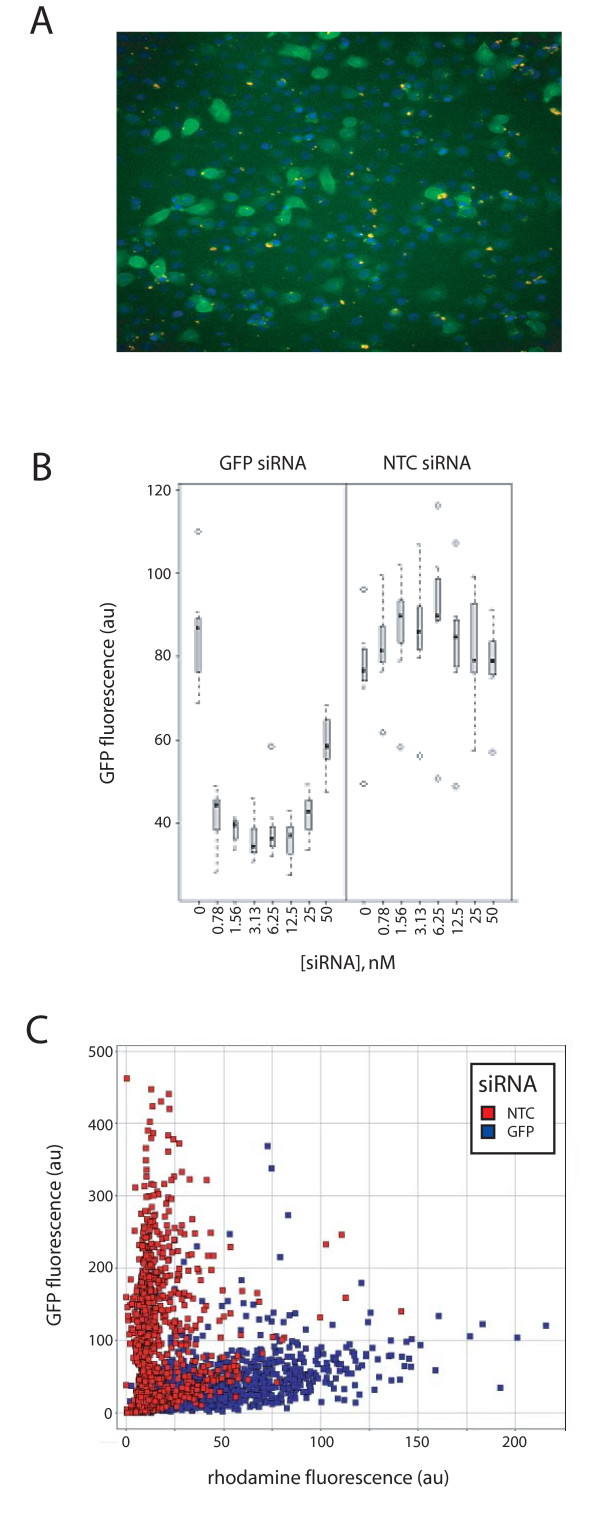
**Single cell analysis of siRNA knockdown of GFP**. siRNAs transfected at increasing doses into RWPE-1 cells stably transduced to constitutively express GFP, are correlated with the reduction of GFP expression, as determined by fluorescence intensity. **A**. GFP-siRNA accumulation and correlation with GFP levels observed by fluorescence microscopy. **B**. Average GFP fluorescence levels of wells treated with a GFP-specific siRNA or a non-targeting control siRNA, as indicated. Each box plot displays the median and intrerquartile range of 8 wells. **C**. For the transfection of siRNAs at a concentration of 3.13 nM, the cells of one well are plotted individually for both GFP and rhodamine fluorescence intensities.

The reduction of GFP levels in cells by the transfection of siRNAs targeting the GFP mRNA sequence is a common and robust system for the study of RNAi biology and mechanism [33]. Its intrinsic robustness notwithstanding, a high degree of variability is frequently observed in experiments modulating GFP expression. We have used this system to understand the extent of variability on experimental results by analyzing the knockdown of GFP levels at the whole well and single cell level. A prostate epithelial cell line (RWPE-1) that constitutively expressed GFP was treated with an siRNA that targets GFP. Despite carefully optimizing transfection efficiency, an appreciable level of heterogeneity was evident in the cells transfected with the GFP-targeting siRNA, the samples treated with an non-targeting control siRNA (NTC) and even in untreated samples. In all cases, a high range of GFP expression can be observed, despite clear overall differences in the samples treated with an siRNA that targets GFP. This heterogeneity is evident in the case of cells transfected with a rhodamine-labeled siRNA that targets GFP, shown in Figure 1A. As can be observed in the figure, siRNAs effectively transfected localize near the nucleus in P-bodies [34-36]. In these studies, the siRNA is labeled with rhodamine on the sense strand, which allows uptake to be monitored, but the label itself does not interfere with silencing, at least in part because the label is on the passenger, or non-targeting, strand. Instead, it allows uptake to be quantitated on a per-cell basis. Perinuclear accumulation of the sense strand is frequently observed in cationic liposome-mediated siRNA transfections [37], and its accumulation enables limiting the evaluation of GFP levels to only those cells that had been transfected effectively. Box plots were generated using eight independent transfections for each siRNA concentration, as shown in Figure 1B. More GFP expression remains in this experiment than in GFP knockdown experiments reported by others (which can report greater than 90% reduction in GFP levels, [11,38]), however these studies evaluated the effectiveness of targeting sequences in co-transfection experiments, which limits GFP expression to only those cells transfected with the RNAi reagents. Studies that examine RNAi knockdown in cell lines stably expressing GFP show knockdown levels consistent with the data in Figure 1B[39-41]. Some of the difficulties of working with RNAi can be observed in Figure 1B, where average effects of siRNA treatment are subject to limitations in transfection reagent concentrations. In particular, in the specific conditions as set up in the experiment, the higher concentrations produce a small reduction in functional knockdown. We have observed this in specific combinations of cell type, transfection reagent and conditions. Overall, transfection reagents have limited ranges of optimal effectiveness, but the exact ranges are highly dependent on the configuration of the experiment, including source of the cell line used. As such, each experiment needs to be individually optimized, as factors that limit the effective range can be either toxicity or siRNA:lipid and complex:cell number ratios that result in suboptimal introduction of the siRNA (Lapan, P. Zhang, J., Pan, J. and Haney, S.A., manuscript in preparation). In the results shown here, the higher siRNA levels are changing the siRNA:lipid ratio, which is the most likely source of diminished efficacy at the higher siRNA levels.

To investigate the extent to which transfection and other sources of variability play a role in the analysis of GFP knockdown by an siRNA, we analyzed the same data at the single cell level. The data for one well where the siRNA was transfected at 3.13 nM are presented in Figure 1C. These data are reported as single cell values that correlate the expression of GFP with the amount of siRNA taken up on a per-cell basis for the GFP siRNA, which was labeled with Rhodamine. The siRNA shows a clear ability to reduce GFP levels. It can also be readily observed that the sample treated with the NTC siRNA includes a significant numbers of cells that intrinsically express low levels of GFP. The number of cells that express low levels of GFP in the control sample affects the mean level of GFP for the pool of untreated cells, and therefore, the extent of knockdown of the treated sample. While the effectiveness of the siRNA in reducing GFP levels is scored as roughly 60% using a whole-well analysis, gating on data within GFP-positive regions (analogous to the gating of cell populations in flow cytometry), the experimental effect is 10-fold, or a 90% reduction in high GFP-expressing cells, with 457 GFP expressing cells in the NTC siRNA treated sample, and 48 in the GFP siRNA treated sample. Heterogeneity of GFP expression is observed by other investigators. In particular, it has been noted that a variety of factors contribute to the perception of stochastic effects on protein expression levels when individual cells are examined. These effects contribute to the observed variability in lines developed from clonally expanded isolates [42], and from constitutive promoters [43].

### Intrinsic target protein levels are highly variable on a per cell basis

**Figure 2:**
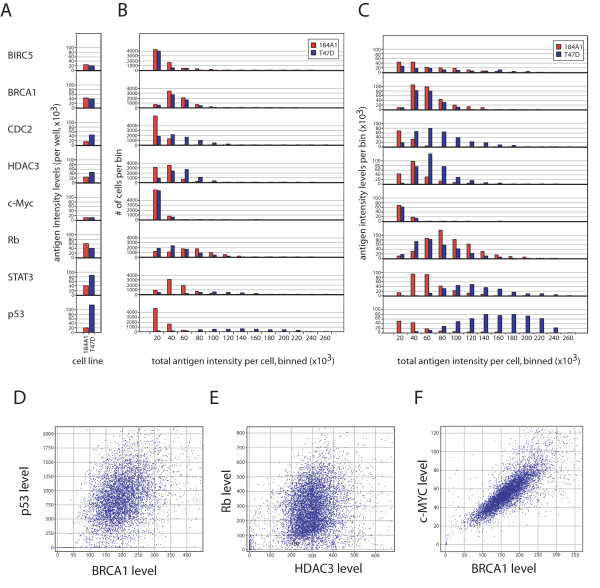
**Wide distributions are observed in endogenous protein levels for cultured cell lines**. Antigen intensity was determined by high content screening following fixation and staining with antibodies that were specific for the indicated protein. Quantitation was achieved by a whole cell mask, which was dilated out from the nuclear region identified by DAPI staining. **A**. Endogenous expression levels of proteins are shown for two breast cell lines, the immortalized line 184B5 and the estrogen-sensitive breast cancer cell line T47D, shown as well or sample mean values of all of the cells. Proteins examined in this study, as indicated in the figure, were quantitated by indirect immunofluorescence and image analysis, and values for each cell are plotted as individual points. All graphs are to the same scale, indicated in the lower left. Approximately 7000 cells were quantitated per sample (antigen/cell line). **B**. Display of sample distributions for the proteins indicated in (A). Data is presented as histograms of cells with increasing levels (total fluorescence intensity, or the sum of all pixel intensities, per cell) of the indicated proteins. **C**. Contribution of cells stratified by antigen intensities on overall abundance measurements. Data is as in panel B, but calculated as the product of the number of cells per bin times the average antigen intensity for that bin. As such, the contribution of each bin to the total well mean response is represented. **D-F**. Correlations between two proteins in cell populations. Protein levels per cell are shown for T47D cells, as indicated in the graphs. Protein levels are mean average fluorescence intensities per cell, as indicated in the axes labels.

Prior to extending the results we observed using siRNA-mediated knockdown of GFP to endogenous proteins, we characterized protein abundance in cell culture populations at the single cell level. This analysis provides a context for understanding how changes in protein levels are measured at the single cell level, and how changes in protein levels affect cellular functions. Eight examples of frequently studied proteins are shown for two breast cell lines in Figure 2. A broad distribution is observed for the proteins indicated in the figure, as well as in other cell lines, including cell lines derived from human tumors (including MCF-7, MDA-MB-235, LnCaP, DU-145, and DLD-1), and epithelial cells that have been immortalized (including the prostate line RWPE-1, the chondrocyte line T/C-28a2 and the breast line 184B5). Proteins we have characterized include transcription factors (STAT1, STAT3, p53, Rb), protein kinases (CDC2, AKT1, ribosomal protein S6 kinase), and other signaling proteins (p16^INK4A^, PTEN). The inherent variability of these proteins is greater than what can be linked to changes resulting from changes in proliferation rates or the cell cycle (as determined by DNA content per cell). Average protein intensities for the indicated proteins are shown in Figure 2A. These data are reported in a manner similar to common methods for describing protein levels in cell lines (e.g. Western blotting and ELISA assays). The data shows that for many of the proteins, levels are higher in the cancer line T47D than they are for the immortalized breast line 184A1, particularly CDC2, STAT3 and p53. Such differences are frequently used to distinguish immortalized breast lines as being "normal," although numerous studies have shown that such immortalized lines bear significant similarities to breast cancer cell lines at the phenotypic and transcriptional levels [44,45]. As such, increased similar levels of BIRC5, BRCA1 and c-Myc between the two lines are consistent with previous studies from this [46] and other laboratories [47,48] that these proteins are significantly affected by immortalization in breast cell lines. Of relevance to the current discussion, different protein levels in the immortalized and cancer cell lines do not exist as discrete examples of cells with high and low levels of a particular protein, but as broad and overlapping ranges of protein levels on a per cell basis (Figure 2B). The increased average levels of such proteins are reflected in these distributions, creating a significant "weighting" of the cells with higher abundances, as shown in Figure 2C (e.g. HDAC3 and p53), while at the same time including a portion of the sample with lower levels. Such broad distributions bear an impact on drug development, as these "side populations" for proteins involved in the cell cycle or DNA damage response may represent cells that are particularly important to disease progression. Subpopulations of cells may be more resistant to chemotherapeutics at the low end of antigen intensity, and may have little contribution to disease progression due to excessive stress and an increased proportion of dying cells at the high end. In such cases, focusing on the disease-relevant populations will have an important benefit to drug development.

We have examined the variability in intrinsic protein levels in cells, including a potential role for bias during the fixation and staining process, by dual-color staining (Figure 2D–2F). We observe that for many pairs, the extent of covariation is low, as observed for p53 and BRCA1 (r = 0.379) and Rb and HDAC3 levels (r = 0.353) in T47D cells. These data indicate that fixation and permeabilization do not play dominant roles in the distribution of antigen intensity. We do observe a higher correlation between c-MYC and BRCA1 levels (r = 0.814), in this particular case, the co-variation may reflect a biological correlation. In addition to the analytical comparison of co-staining patterns, we have examined several pairs of antigen staining to determine whether the staining patterns themselves are independent in cases where abundances are independent, by high-resolution confocal microscopy (results not shown). We find that in cases where two antigens are characterized in the same cells, the patterns are consistent for each antigen, regardless the level of staining for the second antigen. For example, the extent of nuclear staining and the degree of punctate staining observed were independent for the pairs examined (pairwise combinations of HDAC3, Rb and p53), further indicating that artifactual factors, such as uneven permeabilization or fixation, are not the cause of the wide range in antigen levels observed for these cells.

### RNAi-mediated knockdown of PTEN affects phosho-S6 levels

**Figure 3:**
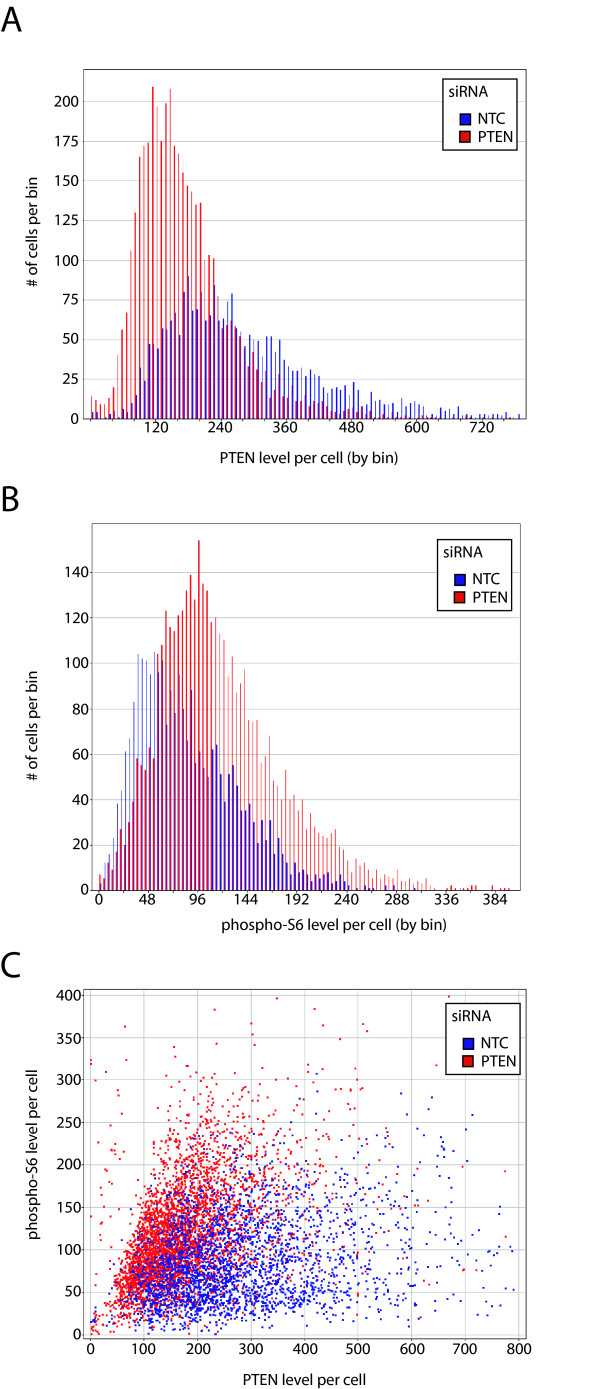
**siRNA-mediated knockdown of PTEN and its effect on phosphorylation of ribosomal protein S6**. The breast carcinoma cell line MDA-MB-231 was treated with siRNAs to characterize the correlation between PTEN levels and ribosomal protein S6 phosphorylation levels. ~15,000 cells are presented. **A**. PTEN levels displayed as a histogram for samples treated with an siRNA targeting PTEN or a non-targeting control, as indicated. **B**. Ribosomal protein S6 phosphorylation for the same cells as in A, shown as a histogram of phosphorylation levels as reported by immunofluorescence intensities. **C**. Pairwise correlation for PTEN and phospho-S6 levels at the single cell level for data presented in A and B.

The regulation of the AKT/mTor pathway represents several important and clinically relevant targets, particularly the inhibition of mTor through rapamycin-related compounds such as temsirolimus [49,50]. The relationship between sensitivity to temsirolimus, PTEN status and phospho-S6 levels have been studied closely for both pharmacogenomic indicators that can be used in patient selection, and in the case of phospho-S6 levels, as a phamacodynamic marker that can be used in drug dosing [51,52]. However, PTEN is only one contributor to activation of the AKT/mTor pathway. This is true in cell culture systems as well as in human tumor samples. We were interested in whether the analysis of RNAi knockdown of PTEN at the single cell level could elaborate on the relationship between its levels and activation of the AKT/mTor pathway. Phosphorylation of S6 is highly sensitive to the activation state of the pathway, both in cellular systems and clinically, where it is a validated biomarker of increased PI3K activity and is correlated with PTEN status. Knockdown results for PTEN are shown at the single cell level in Figure 3A. Testing a range of transfection conditions for PTEN knockdown (similar to Figure 1A) shows that this system is more robust to higher lipid concentrations that is observed for the immortalized chondrocyte line used in the GFP expression studies. The effect of PTEN depletion on pS6 phosphorylation is shown in Figure 3B, where the population of cells treated with the PTEN siRNA shows higher levels of pS6 phosphorylation. In Figure 3C, the levels of PTEN and phospho-S6 are compared for the same samples. The reduction of PTEN level and increase in phospho-S6 levels observed above can be seen as a shift in the PTEN siRNA treated sample.

Figure 3C also shows the complexity of the AKT/mTor pathway when each sample is examined at the single cell level. That is to say, the effect observed in the whole well analyses, a decrease in PTEN results in an increase in phospho-S6 levels, would be expected to cause a negative correlation between these two proteins at the single cell level. Instead, a moderate positive correlation is observed, similar to the correlation observed in the unperturbed endogenous protein levels studied in Figure 2. Although often depicted as a linear pathway that leads to the activation of transcription, translation and metabolic activity, this pathway is under multiple levels of positive and negative feedback regulation of PI-3 kinase, AKT, mTor and ribosomal protein S6 kinase [53-55], which complicates strict correlations between any two points that are separated by one or more of these additional regulatory channels (discussed below). The extensive number of interactions between the AKT/mTor pathway and other regulatory pathways means that cells in culture are in a large number of discrete states. This has been observed elsewhere by our laboratory [56], and has been noted as a complicating factor in therapies that target this pathway, including those that target Her-2^NEU^, PI3K and ERK [57,58]. The use of single cell analysis to track multiple signaling states presents a valuable advance in the study of current and novel theapeutics.

### Defining the role of STAT3 in colon carcinoma growth and survival by single cell analysis of RNAi-mediated reduction in STAT3 levels

**Figure 4:**
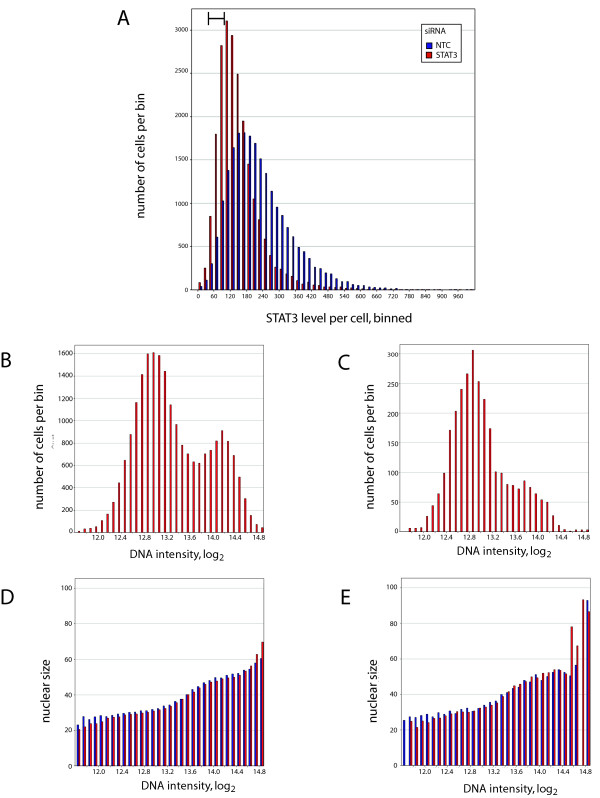
**siRNA mediated knockdown of STAT3**. **A**. Histogram of STAT3 levels in SW480 colon carcinoma cells treated with STAT3 and NTC (non-targeting) siRNAs. Red bars denote STAT3 siRNA treated cells and blue bars represent NTC treated cells. Data presents ~22,000 cells for samples treated with STAT3 and NTC siRNAs each. A region of low-STAT3 expressing cells examined in panels (C) and (E) is indicated in the panel (top left corner). **B**. DNA histogram of cells treated with the STAT3 siRNA. **C**. DNA histogram of low-STAT3 expressing cells (cells are highlighted in panel A). **D**. Nuclear size as a function of DNA content for the entire dataset. **E**. Nuclear size as a function of DNA content for the low-STAT3 cells highlighted in part A, for both STAT3 and NTC treated cells. The measure of DNA content for panels B-E are identical, and therefore the comparison of nuclear size as a function of DNA content may be made directly to the fraction of cells in each phase of the cell cycle (panels C and D, respectively). Color schemes for panels D and E are as in A.

To further investigate the contribution of single cell analysis to cellular signaling studies, we turned to a less complex signaling pathway, the role of STAT3 in cancer cell proliferation and apoptosis suppression. Two examples are shown in Figure 4. In Figure 4A, knockdown of STAT3 in SW480 colon carcinoma cells are shown at the single cell level. Knockdown of STAT3 at the protein level is about 30%, based on average values for replicate wells (3 for each condition, data not shown). Although weakly separated when analyzed at the whole well level, the single cell distributions show a clear effect of treating with the STAT3 siRNA; a K-S test (the Kolmogorov-Smirnov statistic, [29,59]) shows a difference of 0.349 (p < 2.2e-16). Such reductions are typically too small to produce robust phenotypic differences in most whole-well assay formats. There are likely to be many cases where this is correct, but Figure 4A provides a different perspective that more accurately states the situation. It is clear that distribution of STAT3 levels in SW480 cells is too wide for an average reduction of 30% to effectively demonstrate a phenotype associated with STAT3 levels at the whole well level. The overall reduction can be observed in the shift of the distributions, but residual overlap is greater than 50%. If a 30% reduction in STAT3 level does in fact have an effect on these cells, an average change of 30% of STAT3 levels in these samples may not show such an effect because of the wide range in each sample.

While strong changes in average protein levels are required for experiments at the whole well level, analysis at the single cell level shows that STAT3 levels vary over a broad range under both control and STAT3 siRNA treatments. As such, comparisons between low and high STAT3 levels can be made by single cell analysis in cases where whole well differences are less dramatic. As an example, the effect of reducing STAT3 levels by RNAi can be analyzed in the experiment shown in Figure 4. Specifically, STAT3 is constitutively activated in many cancer cell lines, and reduction in STAT3 levels or activity (through RNAi or inhibitors of the JAK/STAT pathways) have been shown to result in growth arrest and apoptosis [60-62].

Proliferation inhibition is the result of the essential role of the protein in growth, but the induction of apoptosis or other forms of cell death has been ascribed to more complex interactions, such as oncogene dependency [63] or oncogenic shock [64]. In these models, cancer cell death results from a release in apoptosis suppression mediated by the signal transduction pathway. The data in Figure 4A can be used to determine whether reducing STAT3 levels through RNAi results in a change in cell health that is distinct from cells with equivalent levels of STAT3 as a result of expression adjustments made during growth in standard culture conditions. This was done through comparing the distribution of cells through the cell cycle in the entire dataset versus a subset of cells where STAT3 levels were low in the STAT3 siRNA-treated sample. For the cells treated with the STAT3 siRNA, 22034 cells were analyzed in the complete dataset and 5471 cells were analyzed in the low-STAT3 population, as indicated in the annotation of Figure 4A. Samples were initially compared for DNA content, as a measure of cell cycle distributions. The data for the entire STAT3 siRNA-treated sample is shown in Figure 4B, and that for the low-STAT3 subset are shown in Figure 4C.

The data in Figure 4B shows that the cells are proliferating, with a significant number of cells in the G2/M phases of the cell cycle. For the low STAT3-containing cells (Figure 4C), the distribution shows a reduction in cells in these phases of the cell cycle, and a majority of the cells in G1. The cell cycle distribution is similar for the low-STAT3 cells of the NTC treated samples, but there are fewer cells and the histogram is not as smooth (not shown). Looking at subgroups with higher levels of STAT3, the proportion of cells in G2 increases somewhat.

In addition to measuring the effect on the cell cycle, the effect of lowering STAT3 levels through RNAi on cell stress and cell death can be determined as well. In this case, such effects would indicate a dependence on high STAT3 levels for survival, either through oncogene addiction or oncogenic shock, two models derived from observations that reduction in oncogene activity can induce cell death. Severe cell stress and cell death are manifest in several ways, including changes to the chromatin and nuclei [28,65-67], which can be quantitated in image-based assays. In the present example, an effect of lowering STAT3 levels on viability would manifest itself as a change in nuclear size in the STAT3 siRNA-treated cells as compared to the NTC siRNA-treated cells. This has been noted in cytometry-based profiling studies [28,68,69], and is shown for SW480 colon carcinoma cells as a function of etoposide treatment in Additional File 1 (details are provided in the Methods section). Nuclear size as a function of DNA content is shown in Figure 4D and Figure 4E for the entire dataset and for the low STAT3-expressing fraction of cells, respectively. Nuclear size increases as a function of DNA content through the cell cycle, as shown for both panels, with increasing nuclear size as cells progress into S phase and again in late G2, immediately prior to anaphase. For the data shown in Figure 4, the relationship between DNA content and nuclear size is essentially identical for the NTC siRNA-treated sample (in blue) and the STAT3 siRNA-treated sample (in red) in both analyses, indicating that cells that have had STAT3 levels reduced through RNAi treatment are not undergoing cell death to a greater extent than control cells. If STAT3 levels were critical to the suppression of apoptosis or necrosis, the nuclear diameter of the cells with low STAT3 abundance would change, relative to the control cells. They would increase in size as a general function of cell stress [27-29], but would typically shrink and become more variegated in classical apoptosis [65,70]. None of these changes are observed in any of the subsets. Taken together, these results suggest that STAT3 is playing an important role in the proliferation of SW480 cells, but is not acting as an essential oncogene through the suppression of apoptosis or necrosis, as would be evident if the nuclei were significantly different.

### p53 dependence on adriamycin sensitivity can be observed following p53 knockdown at the whole well level, and in naturally-occurring low p53-expressing cells at the single cell level

**Figure 5:**
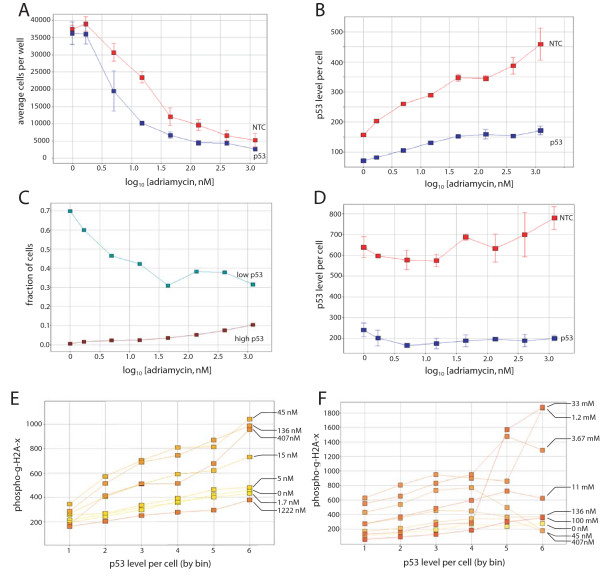
**Dependence of p53 on the response to adriamycin is observed in both p53 siRNA treated cells and in untreated cells with low levels of p53**. **A**. DLD-1 colon carcinoma cells treated with increasing doses of adriamycin as indicated in the figure. Cells were treated with an siRNA targeting GFP (blue) or an NTC (red). Cells not treated with adriamycin were treated with DMSO. **B**. p53 levels following siRNA treatment. An siRNA that targets p53 (blue) or a non-targeting control (red), are shown. siRNA treatments as described in A. **C**. The fraction of cells within each concentration of adriamycin for the NTC treated sample is shown. The fractions of cells with the highest and lowest 20% of the range of p53 levels (burgundy and teal, respectively) in the untreated sample are shown at each concentration of adriamycin. The range of p53 levels in each bin is 0–200 FU for the lowest bin and 800–1000 FU for the highest bin. **D**. p53 levels following p53 or NTC siRNA treatment for 48 hours, and adriamycin treatment for 6 hours. siRNA treatments as described in A **E**. Levels of γ-phosphorylated histone H2A-x levels as a function of p53 levels per cell and adriamycin treatment for 6 hours. Adriamycin doses are as shown in other panels, in a color range from yellow (no adriamycin) to orange (1.222 μM adriamycin). Each data point represents 200–400 cells. **F**. Same as (E), except cells were not treated with an NTC siRNA. Adriamycin concentrations are indicated in the panel.

As a final example of the value of single cell analysis, we characterized the effect of p53 levels on apoptosis and activation of the DNA damage response. The DNA damaging agent adriamycin is toxic to all cells, but the toxicity is more pronounced when p53 is either not expressed or non-functional [71]. We have looked at the dependence of p53 levels in DLD-1 colon carcinoma cells on adriamycin sensitivity at the single cell level. The sensitivity of p53-depleted cells to adriamycin is shown in Figure 5A, where the number of cells per well is reported as a function of adriamycin concentration and treatment with either an siRNA that targets p53 or a non-targeting control (NTC). Control cells shown as not treated with adriamycin were treated with DMSO at the same concentration as the cells treated with the highest concentration of adriamycin. Confidence limits for the data were 0.021 (standard error of 0.0044) for the NTC treated cells and 0.0053 (standard error of 0.0007) for the p53 siRNA treated cells. The levels of p53 for each sample are shown in Figure 5B. This data shows that transfection of an siRNA targeting p53 reduces p53 levels in DLD-1 cells prior to adriamycin treatment, as well as limiting the ability of these cells to fully recover p53 levels as a function of increasing adriamycin concentrations, despite the fact that the increase in p53 levels following DNA damage occurs through post-translational stabilization of p53 protein.

For the sample treated with the NTC siRNA, the amount of p53 per cell was used to divide the cells into groups, and the fraction of cells for each group as a function of adriamycin concentration is shown in Figure 5C. Cells with high levels of p53 are compared to cells with low levels of p53 for each dose of adriamycin. The data shows that cells expressing low levels of p53 are sharply reduced as adriamycin concentrations increase, and to an extent comparable to the reduction of the total cell numbers. This suggests that cells with low levels of p53 are particularly sensitive to adriamycin treatment. Since p53 levels can rise as a direct result of DNA damage, it is also possible that cells with low levels of p53 initially are actually stabilizing p53 and levels are increasing. Therefore, we sought to resolve these two factors in p53-mediated cell survival mechanisms.

We have addressed the question of whether adriamycin sensitivity is affected by p53 levels at the time of DNA damage by looking at how cells respond to treatment prior to when cell death and increased p53 levels are observed. In Figure 5D, the level of p53 in cells treated with siRNAs targeting p53 and the NTC control are shown for cells treated with increasing concentrations of adriamycin for 6 hours. At this time, we do not observe cell death (as reported by the number of cells per well), or a significant increase in average p53 levels (as shown in the figure). However, DNA damage can be observed in these cells in a dose-dependent manner, as determined by changes in DNA and nuclear morphology (data not shown). We have binned these cells by p53 level for each concentration of adriamycin treatment, and measured the levels of γ-H2A-x phosphorylation for each group, as shown in Figure 5E. Phosphorylation of this variant histone occurs in cells following DNA damage [72] independently of changes in p53 level or modification [72,73]. The data shows that cells with higher levels of p53 show stronger DNA damage responses, as evidenced by increased γ-phosphorylated histone-H2A-x levels. Since these are independent responses to DNA damage, it suggests that cells with higher p53 levels may result from a stronger (or more activated) DNA damage response pathway prior to the onset of DNA damage itself, up until a point where the damage is beyond the ability of the cells to respond effectively (1.2 μM and higher concentrations). At high concentrations, significant cell death is observed for all cells (>85% cell killing), and no differential is observed between untreated cells and those treated with an siRNA targeting p53. At concentrations where the dependence of p53 status on adriamycin sensitivity can be observed, single cell analysis has been able to correlate the extent of the DNA damage response induction with p53 levels in cells where p53 levels have not been altered prior to DNA damage. The same general response can be observed in separate experiments using DLD-1 cells that have not been treated with any siRNA prior to that with adriamycin (i.e. no mock or control siRNA transfection at all), shown in Figure 5F. The cells are somewhat more resistant to adriamycin in general, possibly a result of no treatment with liposomes in a transfection, but the pattern of higher p53 levels correlating with higher DNA damage response is still evident.

## Discussion and conclusion

We have applied the general concept of multiparametric single cell analysis to the use of RNAi, and to the relationship between protein levels and chemotherapeutic response. High Content Screening is becoming an important and general approach to biological and therapeutic studies. In addition to increasing the options available for cell-based assays in general, it is opening up new approaches to biological processes and drug development, such as cytological profiling [28,29,66]. Inherent in the latter approaches is the use of single cell cytometry to analyze complex patterns in cellular responses [27]. We have generalized the use of single cell cytometry in several experimental systems and have found that it generally improves experimental analysis, and in some cases, enables challenging questions to be addressed directly. We have used single cell cytometry to address four biological problems: identifying the relevant cells in a knockdown of GFP, correlating the knockdown of PTEN with the increase in activity of pS6 kinase, the effect of knockdown of STAT3 on proliferation and death of colon carcinoma cells and the relationship between p53 levels and responsiveness to DNA damage (both as manipulated by RNAi and as occur intrinsically through standard cell culture conditions).

For RNAi screening in general, there are two applications of single cell cytometry that are potentially valuable. First is a general analysis of knockdown phenotypes by number of cells showing an altered phenotype, rather than average phenotypic change for the two samples. This approach is more in line with other distribution-based methods such as sectoring samples in flow cytometry, and can present data in more biologically-relevant way than reporting as percent-of-control (discussed below). Rigorous analysis of RNAi screening data is currently challenging [15,74], and would benefit from clearer definitions of what constitutes a hit [9,75]. The second benefit of single cell cytometry is the capacity to score cells as a function of the amount of siRNA effectively introduced in cells, as evidenced by the accumulation of the (non-functional) sense strand in P-bodies following efficient transfection. Transfection of siRNAs are frequently associated with off-target effects [76-78], particularly at concentrations typically used for library-based screening (>20 nM) [79,80]. Off-target effects result in many false positive hits in RNAi screens, and impose a significant burden on the post-screening confirmation phase of a project [81]. Transfection at low concentrations (< 10 nM) has been shown to reduce such artifacts, however library screening is performed with many siRNAs that have not been well-validated, particularly for off-target effects. Library screening typically involves higher concentrations because a productive screen requires that cells be reliably transfected, and some balance between the efficiency of transfection and a lack of specificity can be tolerated in the initial screen [15], as long as an effective strategy exists for demonstrating authentic gene-phenotype connections [81,82]. Therefore, off-target effects resulting from high concentrations of siRNA transfections are a common and perhaps unavoidable complication of running siRNA screens. Reduced off-target effects have been associated with pooling or multiplexing siRNAs, particularly in highly complex pools such as are generated by enzymatic preparation of gene-specific siRNA pools (esiRNAs, [83]), at least in part because the concentration of any single siRNA is low.

Reverse-transfection, including the live cell array [7,84,85], is frequently used in functional screens. This format spots the siRNA (or dsRNA for screens in *Drosophila *cells) onto a surface prior to use with cultured cells, and therefore cells are not transfected at a specific concentration, strictly speaking. Single cell analysis can be readily performed on assays following reverse transfection, since these explicitly require image-based readouts. Selecting a subpopulation with consistent siRNA uptake for each siRNA is computationally intensive, and therefore would be difficult to use directly in the primary screen endpoint, but could be used to analyze data from a primary screen that uses a high content (image-based) assay. The siRNAs need to be labeled directly or co-transfected with a labeled siRNA, in order for siRNA levels to be quantitated. However, the benefit of this is that knockdown phenotypes can be scored for cells within specific thresholds of siRNA accumulation, and these thresholds can be adjusted as the data is reviewed, rather than during image analysis. 

Scoring perturbations by fraction of responding cells (in the case of GFP knockdown at the single cell level) and by response magnitude as a function of target level (such as in the example of DNA damage response as a function of p53 levels) highlight important characteristics of biological samples, particularly in the development of human diseases such as cancer. Clinically important roles are played by minor populations within cell types, such as the growth of solid tumors through tumor-initiating cells (cancer stem cells) and the importance of regions within tumors that control angiogenesis and chemoresistance (the hypoxic core of cells within solid tumors). These properties can be observed in cell culture models, but this differentiation is lost in whole-well methods. Tracking effects of candidate therapeutics among rare cells or cells that have reduced proliferation rates can focus decisions on how well promising a strategy may be by limiting analysis to the cells that play the biggest role in disease progression.

A similar situation occurs with pathway analyses. An assay that measures a change in a complex pathway, such as the PI3K/AKT/mTor pathway, cannot help but exclude important factors that contribute to a diverse set of outputs. This heterogeneity may be as much a part of the discordance between target inhibition and clinical response as widely cited factors, such as tumor heterogeneity as a result of genetic instability. In both cases, variability in the cells that constitute a tumor enable a significant number of cells to escape death. The difference between these two scenarios is that genetic instability suggests a somatic evolutionary process, whereas signaling heterogeneity suggests that insufficient control of the pathway results in escape from a therapeutic. In such cases, single cell analysis could improve the search for combination therapeutic strategies. mTor activity is subject to multiple levels of feedback regulation [86,87] and to cross-talk with other pathways, particularly the influence of amino acid and cellular energy levels on mTor activity [55]. As such these influences would need to be measured in a multiparametric assay system, to track changes between two points in such a complex pathway. Taken together, the results presented here suggest that pathways that are quiescent (such p53 during periods of low DNA damage) or truly linear (such as activation of STAT signaling by JAK kinases) should show correlations between two points at the single cell level. This correlation could be used to validate results from RNAi experiments by providing a separate method of linking protein levels to pathway function.

Studies that integrate complex signaling interactions, as opposed to linear events within single pathways, are at the root of systems biology [31,32], and are better able to characterize pathway states in their biological contexts. Such approaches are being shown to be of direct relevance to signaling in disease biology [25,88]. HCS is a strong complement to flow cytometry as a method of single cell analysis because signaling pathway responses can be integrated with cytological dynamics, and as such will extend systems biology into areas such as cancer cell motility and invasion [27,29,89]. These approaches will lead to more innovative approaches to treating disease [90], including complex molecular studies which can be integrated with genetic and epidemiological studies that show subtle but important interactions between common disease loci.

## Methods

### Cell lines, cell culture and reagents

Immortalized breast cell lines 184A1, and 184B5 were generously provided by Martha Stampfer (LBNL, Berkeley, CA). The C19 derivative of T/C-28a2 was developed and generously provided by Manas Majumdar (Wyeth Research, Cambridge, MA). MCF-7, T47D, MDA-MB-235, DLD-1, RWPE-1 were obtained from ATCC (Mannasas, VA). RWPE-1-GFP was developed by transduction of a lentivirus that encodes the GFP gene under the control of the CMV promoter. Media used for each cell line were according to instructions from the source.

Antibodies against γ-phosphorylated histone H2A-x, were obtained from Upstate Biotechnologies (Lake Placid, NY); antibodies against caspase-cleaved PARP and p53 were obtained from Cell Signaling Technologies (Beverly, MA). Fluorescent probes, including DAPI, and antibodies conjugated to Alexa dyes, were obtained from Molecular Probes/Invitrogen (Carlesbad, CA). Adriamycin, 16% paraformaldehyde, and Tween-20 were obtained from Sigma, Inc. (St. Louis, MO). siRNAs targeting p53 were obtained from Ambion, Inc (Austin, TX). Custom synthesized and unmodified siRNAs targeting GFP were obtained from Qiagen (Valencia, CA).

### siRNA transfections

siRNAs were transfected as complexes with cationic liposomes from one of several manufacturers. For each experiment 3–5 commercially-available lipids were tested in a series of concentrations and siRNA:lipid ratios, according to manufacturers instructions. Transfections were four hours long and terminated by a change in media. For each cell line used in each experiment, the optimal lipid and siRNA:lipid ratios were determined using a test siRNA that targets GAPDH and GAPDH enzyme activity was measured for each condition, using the KD Alert kit from Ambion (Austin, TX). Optimal conditions were chosen as those that gave the greatest reduction in GAPDH activity when treated with the GAPDH-targeting siRNA, but minimal toxicity as identified by the NTC siRNA. Optimal conditions for each experiment are listed in Additional File 2.

### Quantitative immunofluorescence

Cells labeled as described in the figures were fixed with 4% paraformaldehyde, washed, permeabilized with 0.2% Triton X-100 and stained with 300 nM DAPI, primary and secondary antibodies and washed again. Antibodies were titrated for optimal imaging, and the lowest concentration that gave a highly-specific labeling of the antigen was used. Sources, dilution levels and fluorescence conditions are listed in Additional File 2.

Antigen intensities and localizations within cells following fixation and staining were imaged using an ArrayScan V^TI ^(Cellomics, Pittsburgh, PA), using a 20 × 0.63 NA objective. Images were analyzed using the Target Activation and Compartmental Analysis image analysis applications from Cellomics. Cellular imaging was accomplished by first locating cell nuclei using DAPI-chromatin fluorescence and expanding the diameter of the nuclei to encompass the cytoplasmic region. Specific adjustments are required for each cell line. Cytoplasmic regions of neighboring cells were optimized in an iterative cycle of algorithm modifications and testing. Fluorescence intensity was captured and interpreted by one of several methods, typically mean fluorescence intensity per cell. Fluorescence measurements were well within the linear range of the image capture system (illumination, light filtering and detection using a cooled-CCD camera), so relative changes in protein levels could be made using relative changes in fluorescence between cells and samples. Non-specific detection is low, as shown in Additional File 3, and this enabled relative changes in protein levels to be determined from the fluorescence intensities.

Nuclear morphology was used as an indicator of cell health. Specifically, changes in nuclear area are indicative of severe cell stresses that result in necrosis or apoptosis. The identification of cells lethally treated with etoposide using nuclear area as an indicator of imminent cell death has been used by several laboratories in both classical apoptosis studies without the use of automation and in cytological profiling approaches. The change in nuclear area following treatment with an inducer of apoptosis is shown in Additional File 1. SW480 cells were treated with 5 μM etoposide for 24 hr, fixed and stained as described above. Cells treated with 10 μM and 20 μM etoposide showed similar distributions of nuclear area.

### Quantitation and statistical analyses

We have used HCS to examine protein levels within cells, and how these levels are manipulated by RNAi, at the single cell level. Data extraction and processing were performed using the statistical programming language R . Data from individual cells were extracted directly from the Cellomics' STORE database using a custom R function getCellData(), which uses a SQL query provided by Cellomics. The getCellData() function allows single cell data to be queried by well, row, column, or plate, one feature at a time, and is described in Additional File 4.

R scripts utilizing the getCellData() function are executed on a LINUX cluster. An auxiliary text file lists the plates and wells to be extracted, as well as the annotation associated with each well. The R script reads the auxiliary file 1nd replicates and merges the annotation with the single cell data as it is extracted from the database. Averaging, normalizations, and transformations are performed in R prior to export as a flat text file. Data is visualized either directly in R or imported into Spotfire for interactive analysis.

## Authors' contributions

PL codeveloped the data extraction and single cell analysis pipeline, and conducted the GFP knockdown experiments, analyzed data, and developed the manuscript; JZ conducted and analyzed the p53 knockdown experiments; JP conducted the antigen distribution, PTEN and STAT3 experiments; AH codeveloped the data extraction and single cell analysis pipeline; SH analyzed data and wrote the draft. All authors contributed to interpretation of data and manuscript revisions.

## Supplementary Material

Additional file 1 Supplementary Figure 1 Effect of etoposide treatment on nuclear area of SW 480 colon carcinoma cells. SW480 cells were plated in a 96-well microtiter plate and cultured for 24 hr, at which time they were treated with 5 mM etoposide (shown in red) or a vehicle control (shown in blue). Nuclear size for each cell is shown as a histogram of the entire dataset after binning as shown in the figure. Click here for file

Additional file 2 Immunofluorescence and siRNA transfection conditions. Specific catalog and treatment conditions for siRNAs, transfection reagents and immunofluorescence microscopy. Click here for file

Additional file 3 Supplementary Figure 2 Representative specific and non-specific staining intensities for the primary and secondary antibodies. Box plots of antigen levels as detected by high content screening. Specific antigens were detected using antibodies as indicated in the panel and non-specific background staining was detected using specific isotypes, is indicated as well. IgG is from rabbit, IgG1 and IgG2a are from mouse. Click here for file

Additional file 4 Extracting high content single cell data for analysis. SWEAVE document describing the routine used to retrieve single cell data from the database. Click here for file
