# Supplementary material for: Single cell cytometry of protein function in RNAi treated cells and in native populations
Source: BMC Cell Biol. 2008 Aug 1;9:43. doi: 10.1186/1471-2121-9-43 (PMC2529295; doi:10.1186/1471-2121-9-43)
Supplement: Additional file 4 — Extracting high content single cell data for analysis. SWEAVE document describing the routine used to retrieve single cell data from the database. [file 1471-2121-9-43-S4.pdf]

Extracting High Content Single Cell Data for Analysis

Peter LaPan, Andrew Hill

July 25, 2008

This document describes the process of directly extracting single cell data from the Cellomics STORE data repository independent of the vHCS:View application. In the initial step a structured query language (SQL) query is used to retrieve cell-level feature data from our Cellomics repository. For convenience, this query was wrapped in a R function called getCellData.

```
getCellData <-  function( plateID="AS_VTI_Z4_050321100001",
                         featureName="MorphologyV2Cell:AreaCh1",
                         Row="A", Col=1) {
    # plateID: Unique Plate ID from Well table aka Unique Plate Descriptor
    # featureName: a character string or character vector containing feature Names
    # Row = uppercase character A-Z indicating Row
    # Col = integer 1-24 indicating Column
    # Note Row and Column are converted to STORE coordinates (zero
    # based integer offsets for both row and col)

    if( Row != "all") {
        if (is.real(Row) | !any(is.element(LETTERS, Row))) {
            stop("Row must be a character between A-Z")
        }
        integerRow <- which(is.element(LETTERS, Row))-1
    } else {
        integerRow <- Row
    }
    if (Col != "all") {
        if (is.character(Col) | Col<1 | Col>24) {
            stop("Col must be an Integer between 1 and 24")
        }
        integerCol <- Col - 1
    } else {
        integerCol <- Col
    }
```

```
    cmd <- paste("perl", "/cenas1/home/plapan/QuerySTORE.pl")
    sql <- "/cenas1/home/ahill/daclust/hcs/sql/get_readout_by_plate_well_feature_template
    if (length(featureName)>1) {
        featureName <- paste(featureName,collapse=",")
        print(featureName)
    }
    cmdLine <- paste( cmd, sql, plateID, featureName, integerRow, integerCol)
    out <- read.table( pipe(cmdLine), sep="\t", header=T)
    if (is.logical(out$ROW)) {
        out$ROW <- substr(as.character(out$ROW),1,1) # suppress F/T - logical conversion
    }
    out

}
```

The R function calls a Perl script that in turn uses the Perl DBI module to run the query. The getCellData function takes as arguments a single unique plate descriptor (UPD), a character vector containing one or more Cellomics Bioapplication feature names (for example, ”MorphologyV2Cell:AreaCh1”), and desired Row and Column identifiers (for example, Row = ”A” and Col = 1, for Well A01). Given these arguments, the getCellData function retrieves the corresponding cell-level data and returns a data frame with one row per cell-feature, and the following columns:

```
PLATEID: the STORE unique plate descriptor
WELLFIELDID: the STORE Well-Field unique identifier
ROW: the well Row (for a 384 well plate, a letter from A-P)
COL: the well Column (for a 384 well plate, an integer from 1-24)
WELL: the well label (zero-padded, for example A01)
CELLID: the STORE unique cell identifier
CELLNUMBER: the cell index within a field
FIELD: the field index within a well
FEATUREID: the STORE unique feature identifier
FEATUREVALUE: the value of the feature readout
FEATURENAME: the name of the Cellomics Bioapplication cell-level feature
                             (for example, MorphologyV2Cell:AreaCh1)
```

Begin by sourcing the getCellData() function. Read in the table of wells which will be assigned to the pinfo variable. Create an output file name.

```
> rm(list = ls(all = TRUE))
> source("/cenas1/home/ahill/daclust/hcs/sql/getCellData.R")
> filename <- "/cenas1/home/plapan/2008/singlecell/jz080226/pinfo.txt"
```

```
> pinfo <- read.table(file = filename, n = -1, sep = "\t", dec = ".",
+     header = TRUE, skip = 0, na.strings = "NA", strip.white = FALSE)
> out.file <- "/cenas1/home/plapan/2008/singlecell/jz080226/jz080226_ss.txt"
```

Look at the pinfo variable which holds one row per well with descriptive annotation:

```
> pinfo
```

```
           ID                       UPD Col Row     sample cellline  Ch1 Ch2
1 JZ012307A498 CEC12303991_080124120001   5   G Adriamycin A498_Vec DAPI GFP
2 JZ012307A498 CEC12303991_080124120001   6   G      LY294 A498_Vec DAPI GFP
  Ch3
1 Cy5
2 Cy5
```

In this table we have the following columns:

```
ID   The plate ID (barcode)
UPD  Universal plate descriptor
col  plate column
row plate row
sample sample information
cellline Cell line information
ch1 Ch1 stain
ch2 Ch2 stain
ch3 Ch3 stain
```

Sample and cellline serve as annotation that will be joined to the data. As many annotation columns as desired can be included. ch1-3 information can also be joined as separate columns, or used to rename the column headings to a more user interpretable value. Separately we need to be aware of the features we need to extract. For this example we will extract:

```
ObjectArea
ObjectTotalIntenCh1
ObjAvgIntenCh1
ObjectVarIntenCh1
TotalIntenCh2
AvgIntenCh2
TotalIntenCh3
AvgIntenCh3
```

Set up a vector to hold all of the feature names to be extracted.

```
> features <- c("TargetActivationV2Cell:ObjectAvgIntenCh1", "TargetActivationV2Cell:Obje
+     "TargetActivationV2Cell:ObjectVarIntenCh1", "TargetActivationV2Cell:AvgIntenCh2",
+     "TargetActivationV2Cell:TotalIntenCh2", "TargetActivationV2Cell:AvgIntenCh3",
+     "TargetActivationV2Cell:TotalIntenCh3", "TargetActivationV2Cell:ObjectArea")
```

Next set up a loop that will read each line of pinfo. Use the UPD row col information to pull up single cell data. Once all data is collected for a well, use nrow to determine the number of cells in the well. Replicate any annotation columns and join. Repeat for each well, i.e. each row of pinfo and stack.

```
> firsttime <- TRUE
> for (i in 1:nrow(pinfo)) {
+     result <- getCellData(pinfo[i, ]$UPD, features, pinfo[i,
+         ]$Row, pinfo[i, ]$Col)
+     sample <- rep(pinfo[i, ]$sample, nrow(result))
+     cellline <- rep(pinfo[i, ]$cellline, nrow(result))
+     result <- cbind(result, sample, cellline)
+     cat(paste(i, "\n"))
+     if (!firsttime) {
+         holder <- rbind(holder, result)
+     }
+     if (firsttime) {
+         holder <- result
+         firsttime <- FALSE
+     }
+     rm(result, sample, cellline)
+ }
```
